# Supplementary material for: Visual Indicator for Intradialytic Hypotension Prediction Using Variation and Compensation of Heart Rate
Source: Diagnostics (Basel). 2024 Nov 26;14(23):2664. doi: 10.3390/diagnostics14232664 (PMC11640372; doi:10.3390/diagnostics14232664)
Supplement: Supplementary file 1 [file diagnostics-14-02664-s001.zip › diagnostics-3259816-supplementary.pdf]

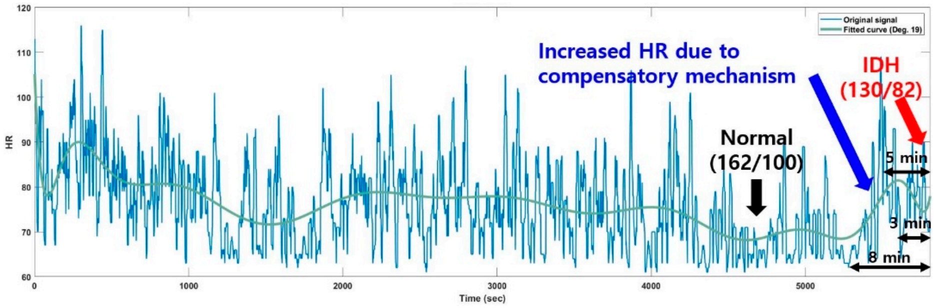

Supplementary Figure S1. HR signal including compensatory mechanisms for patient with IDH.Using the heart rate signal up to 3 minutes before the onset of IDH provides the advantage of incorporating the heart rate increase (which occurs as a compensatory mechanism) into the IDH analysis. However, in this case, the warning time for IDH occurrence is not proactive, which is a disadvantage for responding to IDH effectively.

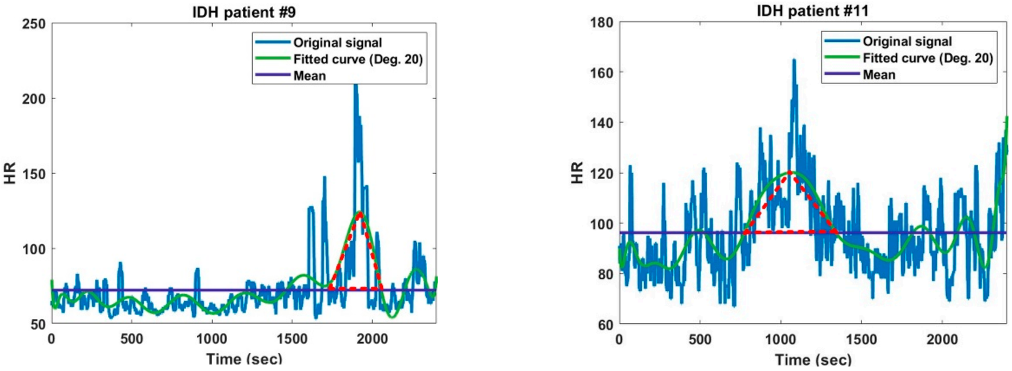

(A) (B)

Supplementary Figure S2. Various duration and peaking issues in the time interval in which the maximum HR area is calculated. IDH cases may have short duration and high kurtosis (Supplementary Figure S2A) or long duration and low kurtosis (Supplementary Figure S2A) in the time interval over which the maximum HR area is calculated.
